# Methodological basics and evolution of the Belgian health interview survey 1997–2008

**DOI:** 10.1186/0778-7367-71-24

**Published:** 2013-09-18

**Authors:** Stefaan Demarest, Johan Van der Heyden, Rana Charafeddine, Sabine Drieskens, Lydia Gisle, Jean Tafforeau

**Affiliations:** 1 Scientific Institute of Public Health, Operational Direction Public Health and Surveillance, Unit Surveys, Lifestyle and Chronic Conditions, Brussels, Belgium

**Keywords:** Health interview survey, Survey-methodology, Fieldwork substitution

## Abstract

**Background:**

The Belgian Health Interview Survey (BHIS) is organised every 4 to 5 years and collects health information from around 10,000 individuals in a face-to-face setting. This manuscript describes the methodological choices made in the sampling design, the outcomes of the previous surveys in terms of participation rates and achieved targets and the factors to be accounted for in data-analysis.

**Methods:**

The BHIS targets all persons residing in Belgium with no restrictions on age or nationality. Trimestral copies of the National Population Registry are used as the sampling frame. To select the respondents, a multistage sampling design is applied involving a geographical stratification, a selection of clusters, a selection of households within each cluster and a selection of respondents within each household. Using matched substitution of non-participating households assures the realisation of the predefined net-sample.

**Results:**

For each BHIS the required number of participants is achieved, including the years when an oversampling of provinces and of the elderly occurred. The sampling design guarantees that the survey is implemented in large cities as well as in small municipalities. A growing problem is related to the sampling frame: it is increasingly subject of deterioration, especially in the Brussels-Capital Region.

**Conclusions:**

The methodological approach developed for the first BHIS proves to be accurate and was kept nearly unchanged throughout the following surveys. Fieldwork substitution contributes to a considerable extent to the success of the fieldwork but yields in higher percentages of non-participation. The sampling design requires special attention when analysing the data: the unequal selection probability, e.g. due to the non-proportional stratification at the regional level, necessitates the use of weights. The BHIS is progressively embedded in the European Health Survey, a process that doesn’t jeopardise the comparability of the Belgian results throughout time.

## Background

The Belgian Health Interview Survey (BHIS) is currently established as the leading health survey in the country with every 4 to 5 years around 10,000 surveyed individuals in some 6,000 households. The survey is carried out by the Operational Direction Public Health and Surveillance of the Scientific Institute of Public Health (WIV-ISP) which provides scientific support for a proactive health policy at the Belgian, European and international levels. The BHIS commenced in 1997 and was re-organised in 2001, 2004 and 2008. The fieldwork of the latest survey started in January 2013. The BHIS is commissioned by all ministers responsible for public health at the federal, regional and community levels.

The purpose of the BHIS is to monitor the health status of the general population as well as health determinants including health behaviours, medical care consumption and social and demographic characteristics [1,2]. The repeated cross-sectional design of the BHIS enables the assessment of health trends and provides evidence for the evaluation of health policy. Throughout the survey years, the content of the survey is increasingly embedded in the approach of the European Health Interview Survey (EHIS). Actually, in the BHIS 2008 several modules of EHIS were already implemented [3].

Data collection is undertaken using face-to-face interviews at the participant’s home. This approach is chosen as it has shown important advantages in comparison with e.g. a mail survey approach (higher response rates) or interviews by telephone (better representativity) [4]. From 1997 to 2008, data were collected using Paper and Pencil Interviewing (PAPI). The interviews are supplemented with a self-administered questionnaire (for the participants aged 15+) covering more sensitive topics like mental health, use of illicit drugs and sexual behaviour.

The analysis and interpretation of the BHIS data require a profound knowledge of the sampling and selection procedures used in the survey and an awareness of the changes that took place throughout the successive surveys. These procedures should guarantee that the results of the BHIS are sufficiently precise and unbiased while taking into account the practical feasibility of the survey given the available resources.

This manuscript describes the methodological choices in the sampling design and in the strategy to select households in the BHIS, the methodological changes since the first survey in 1997, and the outcomes of the previous surveys in terms of participation rates and achieved targets. The manuscript also reflects on how these methodological issues should be considered in the data-analysis.

## Methods

### Target population

The target population of the BHIS consists of all persons with residence in Belgium, including the institutionalised elderly, with no restrictions on age or nationality. The National Population Registry (NPR) is used as the sampling frame. This registry contains information on gender, age, address, citizenship, marital status, etc. of each individual. It is continuously updated based on the information provided by the municipality officials. Indeed, each birth, death and change of address in Belgium has to be declared to the municipality officials. Although the NPR is the most complete and updated population registry in Belgium, using it as a sampling frame implies that those not officially registered (homeless people, unofficial refugees and all those living with them) are excluded from participation in the BHIS. No absolute figures exist on the not officially registered persons in Belgium.

Recent estimations suggest that around 100,000 people are not registered, especially in big cities like Brussels, Antwerp and Gent. A special case concerns the institutionalized people; in the NPR it is mentioned whether someone is institutionalised or not, without defining the kind of institution. Such institution could be a home for the elderly, a convent, a psychiatric institution, a prison… For operational reasons, prisoners and persons living in large convents or in a psychiatric institution are excluded from the survey since this would require a very specific contact procedure (including a permission of organisations’ hierarchy) and adapted interview skills. e People institutionalised in a home for the elderly are included in the survey, given the specific attention of the Commissioners for the health of the elderly population. Therefore all institutionalised people are included in the sampling frame, but their eligibility to participate in the survey is assessed post hoc during the data-collection phase, that is; when the interviewer tries to contact them. In case it turns out that the sampled person lives in a prison, large convent of psychiatric institution, he/she is considered as non-eligible [5].

### Sampling scheme

The BHIS is a cross-sectional household interview survey. Respondents in the BHIS are selected according to a multistage sampling design, involving a geographical stratification, a selection of clusters within each stratum (primary sampling units), a selection of households within each cluster (secondary sampling units) and a selection of individuals within each household (tertiary sampling units). A summary of the sampling scheme applied in the BHIS is presented in Table 1. Belgium consists of three regions: the Flemish Region (around 6.1 million inhabitants), the Walloon Region (3.4 million) and the Brussels-Capital Region (1 million). To obtain accurate estimates for the three regions and, consequently, for the whole country a regional stratification scheme was applied [6], with a sample size of 3,500 interviews in both the Flemish and the Walloon Region and 3,000 in the Brussels-Capital Region. As a consequence, the total basic sample size of the BHIS is set to 10,000 individuals. Within both the Flemish and the Walloon Region, a second stratification is applied at the level of the provinces. The Flemish Region comprises the provinces of Antwerp, Limburg, Flemish Brabant, East Flanders and West Flanders. The Walloon Region is composed of Hainaut, Walloon Brabant, Namur, Liège and Luxembourg. The province of Liège covers a small German Community (around 70,000 inhabitants) which is considered as a separate stratum. The Brussels-Capital Region is not subdivided into provinces. The number of interviews to be realised in every province is proportional to the population size of each province within the region. In the German Community, however, the number of interviews is fixed to 300, as decided upon by the commissioners of the survey. Consequently, the number of interviews to be realised in the other parts of the province of Liège is decreased with this number. As a result, the total number of strata to be considered is 12: the Brussels-Capital Region, the German Community, Liège without the German Community and the 9 other provinces.

**Table 1:** Overview of the sampling scheme of the Belgian health interview survey

	
Overall methodological approach of the BHIS	The aim of the survey is to realise a prefixed number of interviews in every region per quarter. A methodology is used in which groups of 50 individuals (in a number of selected households) will be interviewed. The number of groups equals the prefixed number of interviews in every region divided by 50. In each quarter on average 12.5 individuals per group are to be interviewed. The number of groups to be considered in every province (within every region) is proportional to the number of inhabitants of the provinces.
**Step 1:** selecting municipalities	To determine in which municipalities the groups of individuals will be selected, municipalities are ordered within every province according to their size (number of inhabitants). A systematic selection procedure is used (based on a random start and an interval equal to the size of the province divided by the number of groups to be selected in the province) to attribute groups to municipalities within the provinces. It is possible that several groups are selected in the same large municipality.
**Step 2:** selecting households	Within every selected municipality, households are ordered hierarchically by:
	- statistical sector
	- the size of the household in 5 categories : size 1, 2, 3, 4, and 4+
	- the age of the reference person
	The number of households to be sampled per quarter is theoretically 12,5 divided by the average size of the households of the selected municipality. In order to have enough substitute-households the numerator doubled (25 instead of 12,5). For this calculation, the size of household with more than 4 members is recoded as 4 (because only a maximum of 4 members per household can be selected for the interview).
	The step-size (or 'interval’) used to select the household is defined as the number of households within the municipality divided by the number of household to be sampled in the municipality.
	For every selected household during the sampling, three consecutive households in the order are selected, this in the context of substituting non-participating households. Such quadruples of households are called “clusters”.
	To prevent any order effect, the households within each cluster are randomized, while the clusters themselves are randomised too. After applying this procedure, the fieldwork starts using the first ranked cluster/the first ranked household within the cluster and working from the top to the bottom of the list until the prefixed number of interviews is achieved.
**Step 3:** selecting individuals	In participating households, a maximum of 4 members are selected for the interview: the reference person, the partner (if present) and 3 (no partner) or 2 (partner present) other random selected household members. For non-participating households, substitute households are activated. This process continues till the regional prefixed number of interviews is attainted.

In each stratum, it would have been possible to select the individuals using a random sampling technique. Yet, the travel costs of such scenario are very considerable and exceed the available budget. In this context, it is decided to apply a clustered selection procedure where groups of 50 individuals to be interviewed throughout the year of data-collection are selected from a limited number of municipalities in every stratum. In addition to the practical consideration of the cost reduction, the decision to work with groups of 50 individuals is also based on methodological considerations: this number is judged as the best trade-off that allows to ensure feasibility and a low interviewer-bias.

The selection of the groups and the municipalities is based on a method that combines probability proportional to size (PPS) sampling and systematic sampling. First the number of interviews to be realised in every province is divided by 50 to define the number of groups. The next step involves the ranking of all municipalities according to their population size in every province. A stepwise selection of municipalities is applied using the total population in every province divided by the number of groups as a step size. By doing so, big cities as well as small municipalities can be selected for the survey. In some large cities several groups can be selected.

Given the dynamic nature of the NPR, the data-collection phase is split in four quarters and the quarterly samples do not involve replacement. As a consequence, the number of people to be sampled each quarter per group was (on average) 12.5 individuals. Within each group, households were selected via a systematic sampling procedure: the population registers of the selected municipalities were ordered in terms of statistical sectors (wards), size of the households (1, 2, 3, 4, 4+ members) and the age of the reference person of the household (it is the administrative contact point of a household). The number of households to be selected is determined by dividing 12.5 by the mean household-size in every selected municipality. The total number of households of a selected municipality, divided by the number of households to be selected for the survey in this municipality, provides the selection step.

The last step in the selection process is to identify the members of the households that will be invited to participate. To avoid intra-household correlation and to limit the burden for the households, maximum four household members are selected to participate in the survey. In households with more than 4 members, the reference person and his/her partner are always selected together with two or three other members of the household who have their birthday coming up first after the interview.

An important goal of the BHIS is the assessment of time trends. Therefore no important methodological changes have been introduced since the first survey.

However, two refinements in the survey methodology were applied after 1997: the possibility of oversampling of specific population groups and the geographical division of municipalities with more than one selected group. These changes have no impact on the main methodological approach of the survey. Based on the request of the commissioners, a provincial oversampling was initiated in 2001 to offer provincial health authorities the opportunity to obtain more precise results for their province. The oversampling is subject to payment and the implementation is straightforward. All provinces are informed on the number of sample units they are entitled to according to their population size in the framework of the basic sample. Provinces are then asked if they are interested to inflate their sample size with additional (groups of 50) individuals. These extra numbers are taken into consideration when selecting the groups and municipalities.

Since 2004, and this specifically based on the demand of the Ministry of Social Affairs, the option is also offered to perform an oversampling of specific population groups, particularly the elderly. The operationalisation of this oversampling is more challenging because the sampling approach needs to yield a predefined number of extra elderly while respecting the general principles of the sampling design. This has been resolved through the stratification of the sampling frame in the selected municipalities according to the age of the reference person, and a calculation of the number of households to be sampled in each age stratum, taking into account the estimated age distribution of the household members in the stratum.

In BHIS 1997 and 2001, groups selected from one large municipality, could belong to different statistical sectors. Interviewers were required to contact households throughout the whole territory of the municipality which resulted in supplementary costs. Therefore from 2004 onwards, large municipalities (with several groups) are divided in as many geographical areas as there are groups, ensuring that the population size in each area is more or less equal. In each geographical area, operationalized as a number of adjacent statistical sectors, 50 persons are interviewed. This avoids that an interviewer in charge of one group has to carry out interviews scattered all over the municipality.

Given that the BHIS is not a compulsory survey, it is confronted with non-participation of households, which could be non-contactable households or refusals to participate. To ensure that the predetermined number of interviews is realised in due time, one option would be to increase the sample size based on an assessment of the non-response rate in the country. Yet, when the first edition of the BHIS was carried out in 1997, there was an uncertainty as to the response rate in this survey. Therefore, a decision was reached to apply matched substitution, where for every selected household 3 consecutive households in the ranked list of households used during systematic sampling were selected as substitute-households. The selected household, together with its substitutes is called a cluster. Given the criteria used to rank the households in every municipality, the initial selected household and its substitutes are alike in terms of statistical sector, size of the household and age-group of the reference person. This approach was implemented in the first BHIS and all the subsequent surveys.

The number of clusters is exactly the same as the number of households initially selected for participation. If the first household in the cluster turns out to be a non-participating household, the next household in the cluster will be contacted, in case the second household is a non-participating household, the third household is contacted and so on, until the cluster is exhausted. To ensure that the predetermined number of interviews for every group could be achieved, it was decided to double the number of clusters in every group. This was done by dividing the step size calculated for the systematic sampling of the households by two. In case a cluster is exhausted (all households of the cluster turned out to be non-participants), a substitute cluster is activated and the first household of a new cluster is contacted. Contrary to the households belonging to the first cluster, the households belonging to the substitute clusters are not matched to the initial clusters. In other words, the initial and substitute clusters do not show common characteristics concerning the age of the reference person, the size of the household or the statistical sector.

## Results and discussion

Using the BHIS 2008 as an example, an overview of the distribution of the sample size by province is presented in Table 2. In 2008 a boost of the elderly population of in total 1,250 persons was added to the basic sample, yet this did not alter the basic sampling approach; it just increased the number of groups to be selected. In both the Flemish and the Walloon regions 3,950 interviews, and in the Brussels-Capital Region 3,350 interviews had to be realised. As a consequence, the selection probability differs by region; the relative probability to be selected in the Flemish Region is 0,65, in the Walloon Region it is 1.06 and in the (smallest) Brussels Region it is 3.25. Also differences in provincial sizes resulted in unequal selection probabilities. The oversampling of the German Community, part of the province of Liège, resulted in a low selection probability for the rest of this province. The unequal selection probability affects the representativity of the results, but this is corrected during the estimation process by using sampling weights equal to the inverse of the sampling probability, based on the (known) size of each province-age-household size stratum [7].

**Table 2:** The distribution of the sample size by province, Belgian health interview survey 2008

	**(A)**	**(B)**	**(C)**	**(D)**	**(E)**	(F = (D/A) * 10^**3**^**)**
**Province**	Population^*^	**Fraction (%)**	**Theoretical number of individuals to be interviewed**	**Effective number of individuals to be interviewed (multiple of 50)**	**Number of Groups of 50 individuals**	**The probability for an individual to be selected**
Antwerp	1,700,570	27.7	1098.05	1100	22	0.65
Limburg	820,272	13.2	529.65	550	11	0.67
Flemish Brabant	1,052,467	17	679.57	650	13	0.62
East Flanders	1,398,253	23	902.84	900	18	0.64
West Flanders	1,145,878	19.1	739.89	750	15	0.65
**Total Flemish Region**	**6,117,440**	**100**	**3950**	**3950**	**79**	**0.65**
Hainaut	1,294,844	39.6	1488.6	1500	30	1.16
Walloon Brabant	370,460	10.5	425.89	400	8	1.08
Namur	461,983	13.4	531.11	550	11	1.19
Liège (including GC)	1,047,414	29.1	1204.14	1200	24	
Liège (exluding GC)	973,739			900	18	0.92
German Community	73,675			300	6	4.07
Luxembourg	261,178	7.6	300.26	300	6	1.15
**Total Walloon Region**	**3,435,879**	**100**	**3950**	**3950**	**79**	**1.15**
**Brussels-Capital Region**	**1,031,215**	**100**	**3350**	**3350**	**67**	**3.25**
**Total Belgium**	**10,584,534**	**100**	**11250**	**11250**	**225**	**1.06**

(C) = (3950 * (B))/100 within the Flemish region; (C) = (3950* (B))/100 within the Walloon Region.

Source: Statistics Belgium (population 01.01.2007).

Table 3 presents the evolution of the sample size from the BHIS1997 to the BHIS2008 taking into consideration the oversampling requested by some partners. In the BHIS2001 four of the ten provinces (two in the Walloon region and two in the Flemish region) made use of this possibility. In the BHIS2004 only two provinces financed an oversampling and in the BHIS2008 there were no candidates for oversampling.

**Table 3:** Overview of the sample size of the Belgian health interview surveys1997-2008

**Year**	**1997**	**2001**	**2004**	**2008**
Basic sample	10,000	10,000	10,000	10,000
Provincial oversampling				
Antwerp		350		
Hainaut		500		
Limburg		200	450	
Luxembourg		1,000	897	
Oversampling elderly				
65-85 years			550	
74-85 years				400
85 years +			700	850
Total				
Planned interviews	10,000	12,050	12,597	11,250
Realised interviews	10,221	12,111	12,945	11,254

In addition, an oversampling of the elderly population was done in the BHIS2004 (for the population of 65 years and older) and in the BHIS 2008 (for the population of 75 years and older). The aim of this oversampling was to obtain more precise estimates for the older population in view of the aging of the population. Specific attention was paid to the age group of 85 years and older. Targets were defined by age group. Both the oversampling at provincial level and the oversampling of older people did not affect the representativeness of the results of the BHIS, as post stratification weights are used to calculate regional and national estimates.

**Figure 1:**
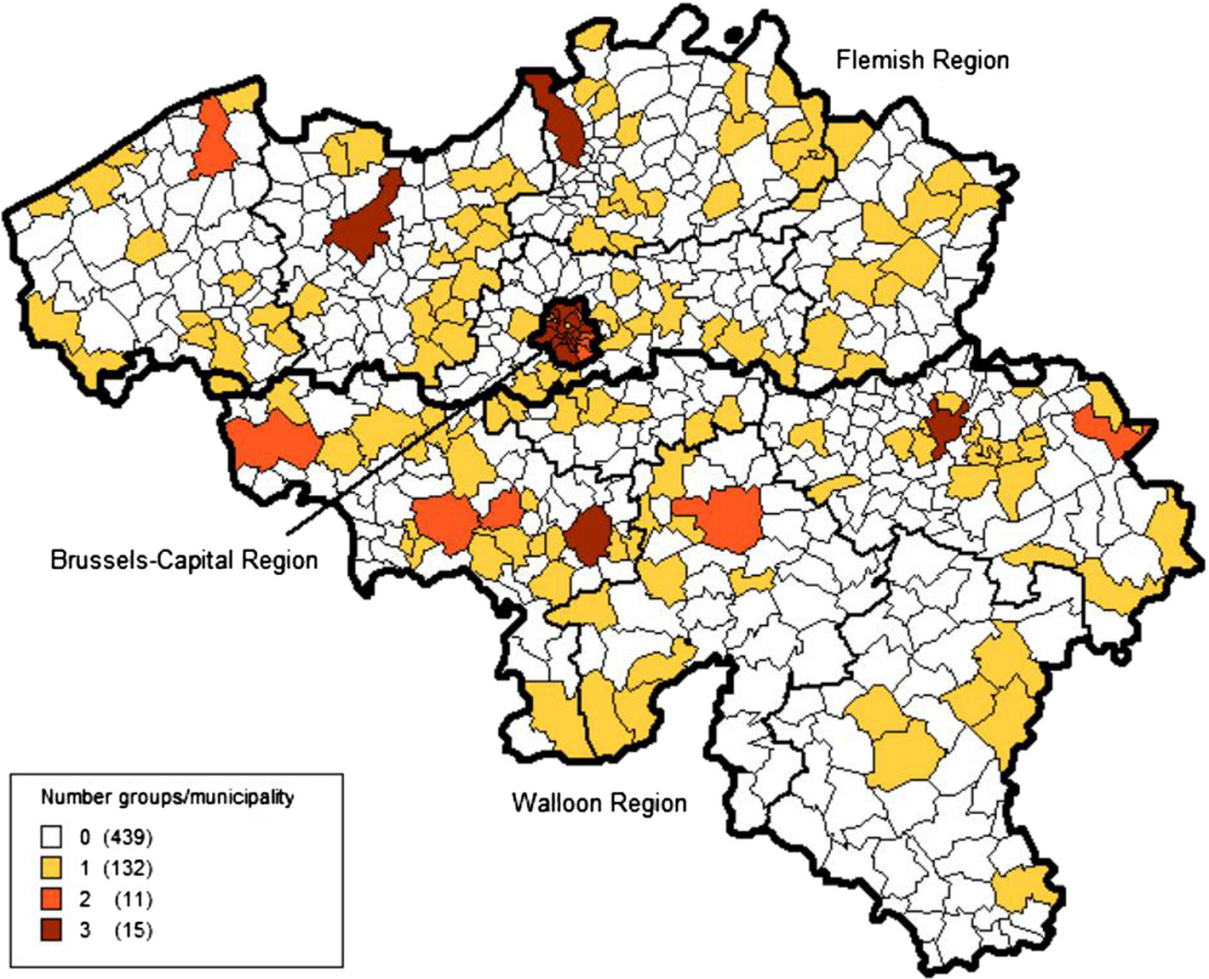
Selected municipalities Belgian Health Interview 2008.

In Figure 1 the geographical dispersion of the municipalities for the BHIS 2008 is presented. All major Belgian cities, but also a number of small municipalities are represented in the sample. In the Brussels Region, all municipalities were selected due to the relative high number of interviews to be obtained in this region. The figure shows also in which municipalities several groups were selected. In these municipalities the statistical sectors were regrouped in geographical areas consisting of a number of adjacent statistical sectors.

The target of the BHIS is defined in terms of a net-sample: the aim is to interview 10,000 individuals (eventually supplemented with boosts of specific sub-populations). Matched substitution is used as a mean to account for non-participation. In Table 4, an overview is presented of the evolution of the participation pattern at the household level throughout the different surveys. In the BHIS 2008, 14,438 households had to be activated to achieve 11,254 individual interviews. Based on the information provided by the interviewers, it was found that for 0.7% of these activated households, the address did not exist, and for 16.1% the household selected for participation did not live (anymore) on the address provided to the interviewers (or a change of address has occurred). In addition, 10% of the households could not be contacted, despite several documented efforts of the interviewers. More than 30% of the households refused to participate, while 40.2% did agree to participate. The figures for the BHIS2008 can be compared with the ones for the BHIS2004 since both included a boost of the elderly populations. While the percentages for non-contactable and refusing households remained stable over both years, the percentage of participating households declined. The steep increase of the number of households that didn’t live (anymore) at the address in the BHIS2008 was mainly due to the delay in the data-collection in the Brussels-Capital Region due to a high drop out of interviewers in the region. This resulted in a deterioration of the sampling frame.

**Table 4:** Participation at household (HH) level, Belgian health interview survey 1997 - 2008

	**1997**		**2001**		**2004**		**2008**	
	**Abs.**	**%**	**Abs.**	**%**	**Abs.**	**%**	**Abs.**	**%**
Non-existing address	***	***	25	0.2	31	0.2	93	0.7
HH doesn’t live at address	***	***	232	2.1	983	7.5	2,328	16.1
Non-contable HH	3,601	31.2	1,978	17.6	1,445	11	1,462	10.1
Refusing HH	3,303	28.6	3,496	31.1	4,107	31.4	4,746	32.9
Participating HH	4,664	31.2	5,533	49	6,530	49.9	5,809	40.2
Invited HH	11,568	100	11,264	100	13,096	100	14,438	100

*** For the BHIS1997, it was not possible to distinguish on-participation due to a non-existing address nor to the fact that the household didn’t live at the indicated address. Such cases were included in the category ’non-contactable households’.

To assess the impact of the substitution procedure, fieldwork data of the BHIS 2001, for which an thorough analysis of the participation pattern was done, are presented in Table 4[8]. All households with correct addresses are subdivided in 4 groups: initially selected households, first, second and third substitutes. Table 5 lists for each group the percentage of contactable versus non-contactable households and, among the contactable households the percentage participating versus refusing households. The percentage of households labelled as 'non-contactable’ grows from 15.7% in initially selected households to 23.5% in the group of third substitutes. Partially this can be due to decreasing efforts of the interviewers to contact already the fourth household to obtain participation. The figures also show a decline in the participation rate; 64.4% of the initial household for which a contact could be obtained, agrees to participate against only half of the contacted third substitutes.

**Table 5:** Overview of the participation status of household selected for participation, Belgian health interview survey 2001

**11,007 valid addresses (100%)**							
Initial selected HH		First substitute		Second substitute		Third substitute	
6,436 (58.5%)		2,775 (25.2%)		1,281 (11.6%)		515 (4.7%)	
Initial selected HH (100%)		First substitute (100%)		Second substitute (100%)		Third substitute (100%)	
Contactable	Non-contactable	Contactable	Non-contactable	Contactable	Non-contactable	Contactable	Non-contactable
5427 (84.3%)	1009 (15.7%)	2207 (79.5%)	568 (20.5%)	1001 (78.1%)	280 (21.9%)	394 (76.5%)	121 (23.5%)
Contactable HH (100%)		Contactable HH (100%)		Contactable HH (100%)		Contactable HH (100%)	
Participation	Refusal	Participation	Refusal	Participation	Refusal	Participation	Refusal
3,493 (64.4%)	1,934 (35.6%)	1,288 (58.4%)	919 (41.6%)	547 (54.6%)	454 (45.4%)	205 (52.0%)	189 (48.0%)
Overall participation: 5,553 (61.3%)				Overall refusal: 3,496 (38.7%)			

Statistical methods for estimating population parameters are based on the assumption that the observations were selected independently and that each observation has the same selection probability. The BHIS approach, in which a stratified clustered sampling procedure is applied, deviates from this assumption: the selected households are clustered geographically (limited number of selected municipalities), and within a participating household only a sub-sample is taken (maximal 4 household members are selected to participate in the survey). Additionally, regional stratification contributes even further to the unequal selection probabilities. Analysing BHIS data has to account for these design effects. Weighting factors are calculated that reflects the differential selection probability, corrects for differential response rates and adjusts the (demographic) sample distribution by using known population distributions. Consequently, the weight for each sampled individual in the BHIS is the product of the reciprocal of the selection probability within a household) and of a post stratification factor for each province according to age, gender, household size and quarter of the year in which the interview was done.

Table 6 presents the BHIS2008 results for the indicator 'subjective health’ with and without taking the design effects into account. Point estimates and standard errors are to a considerable extent influenced by the design effect: the global absolute difference between the estimate without taking the design effect into account and the one calculated after taking the weights, clustering and stratification into account is not less than 3.17%. At the same time, the standard error increases in relative terms with 42%. Therefore an analysis ignoring the design effect would yield biased point estimates that cannot be considered representative for the survey population. Yet, the impact of both stratification and clustering has shown to be quite minimal. This is mainly due to the fact the number of clusters (=households) is big and the number of units (=individuals) within a cluster (=household) is limited (maximum 4).

**Table 6:** Proportion of people in moderate to bad perceived health, by background characteristics

		**Analysis not taking into account the design effects**		**Analysis taking into account the design effect**		**Absolute difference between the two estimates (in%)**	**Increase in standard error when taking into account the design effect (in%)**
		**Estimate**	**Standard error**	**Estimate**	**Standard error**		
Gender							
	Men	23.31	0.71	20.53	0.93	-2.78	30.99%
	Women	29.07	0.71	25.73	0.94	-3.34	32.39%
Age group							
	15-24	8.82	0.94	6.52	1.06	-2.30	12.77%
	25-34	13.05	1.01	11.02	1.14	-2.03	12.87%
	35-44	16.77	1.05	15.95	1.45	-0.82	38.10%
	45-54	26.25	1.27	26.68	1.79	0.43	40.94%
	55-64	30.28	1.36	28.68	1.78	-1.60	30.88%
	65-75	39.24	1.80	39.45	2.52	0.21	40.00%
	75+	48.99	1.39	48.19	2.22	-0.80	59.71%
Educational attainment							
	No diploma/only primary	46.76	1.60	42.59	2.45	-4.17	53.13%
	Lower secondary	37.68	1.38	35.09	2.07	-2.59	50.00%
	Higher secondary	25.17	0.89	22.12	1.15	-3.05	29.21%
	Higher	15.65	0.67	14.30	0.99	-1.35	47.76%
Region							
	Flemish Region	24.15	0.78	21.41	1.00	-2.74	28.21%
	Brussels-Capital Region	26.69	1.00	25.74	1.17	-0.95	17.00%
	Walloon Region	28.77	0.87	26.33	1.11	-2.44	27.59%
Total		26.42	0.50	23.25	0.71	-3.17	42.00%

Impact of the design effects, BHIS; 2008.

## Conclusions

Compared with most other European countries, Belgium has a relatively short history of organising health surveys. The organisation of four BHIS so far, shows that the methodological approach developed in the years preceding the first survey is quite successful. For every survey year, the net-sample at the regional level and consequently at the country level has been obtained. So far, there is no need to adapt fundamentally the methodology applied in the survey. Some minor changes smoothed the data-collection, although some methodological issues remain points of discussion.

From the 2001 survey onwards, prior to the sampling procedure, municipalities for which several groups had to be selected were subdivided in several geographical homogeneous units according to the number of groups. By doing so, the travel time and travel costs for interviewers were set to a minimum. Unfortunately, this approach is only applicable in case several groups are selected in the city. In sparsely populated, large municipalities, interviewers remain confronted with considerable travel distances.

A possible drawback of the complex sampling design, including stratification and clustering at different levels is that point and variance estimates will be biased if design effects are not taken into consideration during data analysis. Although multilevel analysis applied to (continuous and discrete) items of the BHIS1997 to assess the effect and the magnitude of the design showed very little intra-municipality correlation and moderate intra-household correlation [9], there is a need to correct for this correlation when presenting the results. The unequal selection probability, e.g. due to the non-proportional stratification at the regional level, and the oversampling of specific population groups, requires the use of sampling weights. Considering weights and design settings when analysing survey results is essential [10] but in practice not always applied.

The BHIS is focused on the realisation of the fixed number of interviews at the end of the fieldwork-phase. Using field substitution is believed to be the 'engine’ to achieve this. Substitution would also assure that hard-to-reach households (either in terms of 'hard to contact’ or 'hard to participate’) would in the end be represented in the net-sample since hard-to-reach households are to be substituted with 'similar’ households. Nevertheless field substitution remains a contested survey practice [11]. In the European Social Survey, for instance, substitution is simply not allowed as it does not meet the requirements of probability sampling [12,13]. However, Smith has explored the use of substitution in surveys and concluded that optimal substitution (including close field supervision, full-efforts to contact initial cases and substitutes,…) resembles the use of random replicates and can be considered a full-probability design [14].

Although it is assumed in the BHIS that substitution partially prevents a bias that could be introduced due to a practice in which interviewers avoid 'hostile’ areas (since substitution takes place within the original statistical sector) or hard to reach households (since criteria as household size and age of the reference person are used for substitution), analyses on the BHIS2004 results showed no empirical evidence for this assumption [15]. Yet, based on the experience of the BHIS it is felt that substitution positively affects the quality of the data collection in four other ways; (1) It optimises the efforts interviewers will 'invest’ in trying to contact a household (since the substitutes will probably, given the common characteristics with the initial household, be as hard to reach). (2) It assures a better spread of the interviews throughout time. Given the approach to launch a batch of households to be contacted at the start of each trimester, not applying substitution would cause a peak of interviews during the first phase of every trimester. This peak does also exist in the current approach (given that +/- 60% of all participating households are initially selected household) but is smoothed by the substitution process. (3) It facilitates the monitoring of the data collection phase and enables adjustments in the number of interviews to be realised. Although updated versions of the NPR are used to compose the sample, deterioration of their quality is inevitable. Substitution enables to account for this, since it uses factual data (communicated by the interviewers) on the number of respondents. By monitoring the accrual rate per group, per trimester (number of effective interviews), the substitution approach enables the decision to stop the activation of substitute-households once the targets are realised. (4) It is very closely target-oriented, since it does not use estimates for the participation-rate, but is based on the actual number of realised interviews.

Fieldwork substitution has also some setbacks: (1) Although the initial households and the substitutes have some common elements (size, age reference person, statistical sector), their health profile can be significantly different. The assumption that the initial households and the substitutes are 'alike’ can be hampered. (2) Substitution negatively affects the duration of the data collection phase. Since every time substitution is applied, the whole process of inviting households to participate, communicating the (new) addresses to the interviewers, the interviewers’ attempts to contact the households,… has to be repeated, the delay between the activation of the initial household and finally the interview with a substitute-household tends to be substantial. (3) Finally substitution complicates the administrative procedures, since it presumes an individual follow up of every interviewer on a day to day basis in order to activate, or not, a substitute-household.

The finding that the methodological approach applied so far in the BHIS was successful in quantitative terms – the scheduled number of interviews were realised – is no assurance for achieving the goals of the current BHIS2013. For the BHIS2013 a shift was made from a PAPI to a CAPI-application for the face-to-face interviews. This may reduce the response rate in specific population groups (e.g. women and older people) and also affect the responses [16]. If proven to be successful, the use of CAPI will result in a tailored content of (parts of) the questionnaire according to the demands of the different commissioners. Another change in the BHIS2013 is that the data collection has been subcontracted to Statistics Belgium that has integrated the survey in their other surveys (e.g. Labour Force Survey, Survey on Income and Living Conditions). Although the fundamental methodological choices that grounded the BHIS are left untouched (e.g. the application of matched substitution), some practicalities in the data-collection were adapted (e.g. the communication with the interviewers, the documentation of the contact-attempts).

BHIS provides unique data on the health of the inhabitants of the country. The current embedment in EHIS will enable to compare the Belgian results with these from all European countries which implies a major improvement compared with the post-harmonisation process that is needed to enable comparing of European data. Future challenges of the BHIS include the development of a Health Examination Survey (HES) as an expansion to the BHIS approach and the linkage of BHIS data with administrative databases such as health consumption or mortality by cause data. A first attempt to link data of the BHIS2008 with data from the health insurance database is now on-going.

## Competing interests

The authors declared that they have no competing interest.

## Authors’ contributions

SD and JVdH drafted the paper. RC, SDr, LG and JT reviewed and commented the manuscript. All authors approved the final and submitted version. All authors read and approved the final manuscript.

## References

[B1] De Bruin A Picavet HS Nossikov A Health Interview Surveys: towards international harmonization of methods and instruments 1996 Copenhagen: World Health Organisation 8857196

[B2] Van Oyen H Tafforeau J Hermans H Quataert P Schiettecatte E Lebrun L The Belgian health interview survey ArchPublicHealth 1997 55 1 13

[B3] Aromaa A Koponen P Tafforeau J Vermeire C Evaluation of health interview surveys and health examination surveys in the European union Eur J Publ Health 2003 13 67 72 10.1093/eurpub/13.suppl_1.67 14533752

[B4] Van Oyen H Demarest S Tafforeau J Life at risk: lifestyle characteristics in Belgium Am J Epidemiol 1999 149 37

[B5] Van Oyen H The institutionalised populations in health survey.: Paper presented at the United Nations Meeting on Disability Measurement, New York 2001 http://unstats.un.org/unsd/disability/pdfs/ac.81-7-6.pdf

[B6] Quataert P Van Oyen H Tafforeau J Schiettecatte E Lebrun L Bellamammer L Health Interview Survey, 1997. Protocol for the selection of the households and the respondents 1998 Brussel: S.P.H

[B7] Tibaldi F Bruckers L Van Oyen H Van der Heyden J Molenberghs G Statistical software for calculating properly weighted estimates from health interview survey data Soz Praventivmed 2003 48 269 271 10.1007/s00038-003-3017-3 12971115

[B8] Demarest S Van der Heyden J Charafeddine R Tafforeau J Van Oyen H Van Hal G Socio-economic differences in participation of households in a Belgian national health survey Eur J Public Health 2012 10.1093/eurpub/cks158 10.1093/eurpub/cks158 23183496

[B9] Renard D Molenberghs G Van Oyen H Tafforeau J Investigation of the clustering effect in the Belgian health interview survey 1997 Arch Public Health 1998 56 345 361

[B10] Berchtold A Key elements in the statistical analysis of surveys Int J Public Health 2007 52 117 119 10.1007/s00038-007-6081-2 18704291

[B11] David MC Bensink M Higashi H Donald M Alati R Ware RS Monte Carlo simulation of the cost-effectiveness of sample size maintenance programs revealed the need to consider substitution sampling J Clin Epidemiol 2012 68 1200 1211 23017637 10.1016/j.jclinepi.2012.04.013

[B12] Lynn P Häder S Gabler S Laaksonen S Methods for achieving equivalence of samples in cross-national surveys: the European social survey experience Journal of Offical Statistics 2007 23 107 124

[B13] Pickery J Carton A Oversampling in relation to differential regional response rates Survey Research Methods 2008 2 83 92

[B14] Smith TW Notes on the use of substitution in surveys 2007 ISSP unpublished NORC report, Chicago

[B15] Van der Heyden J Demarest S Van Herck K De Bacquer D Tafforeau J Van Oyen H Association between variables used in the field substitution and post stratification adjustment in the Belgian health interview survey and non-response International Journal of Public Health 2013 2013 10.1007/s00038-013-0460-7 10.1007/s00038-013-0460-7 23619721

[B16] Eckholm O Hesse U Norlev J Davidsen M A comparison of CAPI and PAPI in a nationally representative Danish health survey 2004 Europe: European Conference on Quality and Methodology in Official Statistics

